# Exploring barriers to participation and adoption of telehealth and telecare within the Whole System Demonstrator trial: a qualitative study

**DOI:** 10.1186/1472-6963-12-220

**Published:** 2012-07-26

**Authors:** Caroline Sanders, Anne Rogers, Robert Bowen, Peter Bower, Shashivadan Hirani, Martin Cartwright, Ray Fitzpatrick, Martin Knapp, James Barlow, Jane Hendy, Theti Chrysanthaki, Martin Bardsley, Stanton P Newman

**Affiliations:** 1 Health Sciences Research Group (Primary Care), The University of Manchester, 5th Floor, Williamson Building, Oxford Rd, Manchester, UK; 2 Health Service Research, City University, Northampton Square, London, UK; 3 Department of Public Health, University of Oxford, Rosemary Rue Building, Old Road Campus, Headington, Oxford, UK; 4 London School of Economics and Political Science, Houghton Street, London, UK; 5 Imperial College Business School, Exhibition Road, South Kensington Campus, London, UK; 6 Department of Health Care Management and Policy, University of Surrey, Guildford, Surrey, UK; 7 The Nuffield Trust, 59 New Cavendish Street, London, UK; 8 School of Health Sciences, City University, 20 Bartholomew Close, London, UK

**Keywords:** Telehealth, Telecare, Patients’ perspectives, Non-adoption, Non-participation, Barriers, Qualitative research, Whole System Demonstrator

## Abstract

**Background:**

Telehealth (TH) and telecare (TC) interventions are increasingly valued for supporting self-care in ageing populations; however, evaluation studies often report high rates of non-participation that are not well understood. This paper reports from a qualitative study nested within a large randomised controlled trial in the UK: the Whole System Demonstrator (WSD) project. It explores barriers to participation and adoption of TH and TC from the perspective of people who declined to participate or withdrew from the trial.

**Methods:**

Qualitative semi-structured interviews were conducted with 22 people who declined to participate in the trial following explanations of the intervention (n = 19), or who withdrew from the intervention arm (n = 3). Participants were recruited from the four trial groups (with diabetes, chronic obstructive pulmonary disease, heart failure, or social care needs); and all came from the three trial areas (Cornwall, Kent, east London). Observations of home visits where the trial and interventions were first explained were also conducted by shadowing 8 members of health and social care staff visiting 23 people at home. Field notes were made of observational visits and explored alongside interview transcripts to elicit key themes.

**Results:**

Barriers to adoption of TH and TC associated with non-participation and withdrawal from the trial were identified within the following themes: requirements for technical competence and operation of equipment; threats to identity, independence and self-care; expectations and experiences of disruption to services. Respondents held concerns that special skills were needed to operate equipment but these were often based on misunderstandings. Respondents’ views were often explained in terms of potential threats to identity associated with positive ageing and self-reliance, and views that interventions could undermine self-care and coping. Finally, participants were reluctant to risk potentially disruptive changes to existing services that were often highly valued.

**Conclusions:**

These findings regarding perceptions of potential disruption of interventions to identity and services go beyond more common expectations that concerns about privacy and dislike of technology deter uptake. These insights have implications for health and social care staff indicating that more detailed information and time for discussion could be valuable especially on introduction. It seems especially important for potential recipients to have the opportunity to discuss their expectations and such views might usefully feed back into design and implementation.

## Background

Within many healthcare systems, there has been a growing focus on the value of telehealth and telecare interventions for improving quality and cost-effectiveness of care for people with long term complex health and social care needs [1]. Such technologies are varied, but a distinction is generally drawn between ‘telehealth’ and ‘telecare’. Telehealth interventions allow remote exchange of data (e.g. blood glucose and blood pressure readings) and additional information between a patient and health care professional(s) to assist in the diagnosis and management of a health care condition(s). Telecare allows the remote monitoring of changes in an individual’s condition or environment in order to manage the risks of independent living. Examples include sensors to detect movement, falls, flooding and gas [2].

Telehealth and telecare interventions have been evaluated in various international settings. Some evidence of positive outcomes has been reported including improved clinical indicators and reduced health service use [3], as well as enhanced feelings of security and increased satisfaction with health and social care services [4]. However, a number of studies have demonstrated that these types of interventions often fail to be successfully implemented and adopted within routine healthcare [5]. Additionally, evaluation studies and trials have reported recruitment difficulties with up to 80% refusal rate [6]. Survey studies have been able to summarise and quantify some reasons why potential participants refuse to join such trials, with frequent reasons described as participants being too busy, discomfort with the technology, belief that the technology could not help them, and preference for existing services [6-8].

Whilst survey studies have provided figures on categories of reasons for refusal, they are unable to explain responses including *why* people have discomfort with the technology, *why* they believe the technology cannot help them, or *why* they prefer existing healthcare arrangements. These questions can best be addressed using qualitative research methods [9]. Such methods are increasingly being viewed as an important component of trials of complex interventions [10,11] with a number of qualitative and mixed method studies providing important insights regarding factors influencing poor recruitment for trials [12], and more specifically, the ‘non-adoption’ of e-health innovations such as a personal electronic health record [13].

More broadly, there has been an increasing focus on the use of health related technologies in older age, questioning assumptions made about needs, use and phobia of such technologies [14,15]. A number of studies have focused on the use of home-based health related technologies in the context of older age and consider some key issues regarding such interventions which are increasingly being deployed as part of efforts to reduce the burden of long-term care on formal services, by supporting community based self-management. The capacity for specific technologies to promote independence and enhanced self-management is complex. For example, ambivalent ideas about domiciliary oxygen therapy in one study indicated perceptions that it fostered greater independence, but on the other hand there were concerns that the technology could induce dependence on its use [16]. Similar issues were raised by Lehoux et al. who studied four technological health care interventions at home (IV antibiotic therapy, parenteral nutrition, peritoneal dialysis and oxygen therapy). They found that patients were ambivalent about the benefits and drawbacks of technology because whilst technology can be pivotal in making patients autonomous, it also imposes heavy restrictions that are intimately interwoven with the nature of the particular disease and with the patients’ personal life trajectory [17].

Gitlin et al., highlight that acceptability of assistive technologies for older people is dependent on whether the device is perceived to support or undermine their sense of identity [18], and there has been some concern amongst key stakeholders regarding the impact of telecare interventions on autonomy associated with close surveillance [19]. McCreadie and Tinker also stress the importance of researching ‘felt need’ because their respondents were often managing bravely in difficult circumstances but did not see themselves as needing help [20]. These studies raise important issues to consider in exploring the barriers to the use of technologies for supporting self-care including TH and TC which are further explored in this paper.

Within the UK, the 2006 white paper ‘Our health, Our Care, Our Say’ published by the Department of Health focused on telehealth and telecare technologies as *‘pointing the way to the future’* for supporting people with longer-term health and social care needs [21] (p.118). The paper promised a series of demonstrator pilots and a plan *‘to motivate all commissioners to drive services in this direction’* (p.120). The acceptability of these technologies to potential recipients is therefore important for policy-makers and health and social care staff involved in any future roll out. The plans set out in the white paper led to the design and conduct of a large scale randomised controlled trial of telehealth and telecare technologies across the south of England: the Whole System Demonstrator (WSD) evaluation project. This paper reports a nested qualitative study within the WSD programme designed to investigate reasons for declining or withdrawing from the main trial. This became an increasingly salient issue as similar to other evaluation studies, rates of refusal were a concern and early reports highlighted the slow recruitment because of the ‘patient not wanting’ the interventions [22]. Of those who agreed to receive a visit at home to discuss the potential installation of TH or TC equipment (n = 9214), and to be assessed for eligibility to join the trial, 36.7% (n = 3384) did not enter the trial.

A focus on the reasons for non-participation in the trial is important because the problems of non-participation and barriers to adoption of TH and TC have been acknowledged but not yet addressed in published evidence [23]. Additionally, perceived priorities regarding discussions on the potential benefits of remote monitoring are often based on assumptions rather than empirical understanding [24]. Existing research on reasons for non-participation in randomized controlled trials and other research has helped to demonstrate the complex issues regarding recruitment to trials. Such studies have found that common barriers to participation include general concerns with the trial setting and the research process; and more specifically, concerns about randomization [25-27]. In focusing on non-participation and withdrawal from the WSD trial, this paper has some overlaps with the wider literature on non-participation and withdrawal from RCTs. However, the paper mostly reflects the focus of participants on the potential impact of telehealth and telecare if they were to take part in the trial. The latter is illustrated via data presented in subsequent sections of the paper and we return to this point in the final discussion.

## Participants and methods

Participants were recruited from across three sites taking part in the trial: Cornwall, Kent, and the east London borough of Newham. All Primary Care Trusts (PCTs) and Local Authorities within these sites took part in the trial. The randomised controlled trial was a cluster design (reported in detail elsewhere) [2], in which general practices were randomised to receive access to telehealth or telecare for their populations. Additionally, participants randomised to the control arm were offered telehealth or telecare following re-assessment at the end of the 12 month trial. Telehealth eligible patients had one of 3 index conditions: diabetes, chronic obstructive pulmonary disease (COPD), or heart failure (HF). Telecare eligible participants were diverse, but were eligible if assessed as ‘at risk’ for independent living. Telehealth equipment included a monitor unit via which recordings from peripheral devices (measuring blood pressure, blood glucose, blood oxygen level, weight, peak flow) were to be uploaded to a monitoring centre. The peripheral equipment was allocated in various combinations contingent upon initial assessment of need. The monitoring centres prioritised and tailored response according to need based on the information received. Telecare interventions also varied according to assessed need but included various sensors to detect gas, water overflow, falls and movement around the property. Such sensors would trigger alarms direct to a monitoring centre if anything abnormal was detected, allowing emergency intervention.

Following cluster allocation of practices, eligible patients within practices were then approached to take part in the trial and to receive the technology where appropriate. Details of the process of recruitment to the trial are published elsewhere [2,28], but key stages included sending a letter requesting permission from potentially eligible patients to allow data to be passed to the WSD teams (known as the ‘data sharing letter’) so that they could be contacted to discuss eligibility for the interventions and the trial. Where permission was granted, patients were telephoned at home for an initial discussion about the interventions and the trial. They were then asked if they would be willing to receive a home visit to have a more detailed discussion about the interventions and the trial; and to assess eligibility. The home visit was then conducted by members of the implementation teams in the 3 study sites^i^, and here patients were shown information cards with images to describe the equipment that would be offered if they joined the trial and in some cases, they were shown samples of the peripheral telehealth equipment or telecare devices. At this visit, people were asked to sign a consent form if they agreed to take part. If they did not want to consent to join the trial at this visit, staff could then ask them whether they would be willing to receive information about the qualitative study which was aiming to include a number of people who were not taking part in the trial. Consequently, participants for the qualitative study were recruited following the home visit and assessment outlined above.

We initially planned to sample purposefully to aim for maximum variation [29] in terms of gender, age, ethnicity, as well as health and social care needs. We had also planned to sample theoretically, according to iterative analysis of initial interviews. However, circumstances of recruitment necessitated an opportunistic approach. This is because sampling was contingent upon health and social care staff within the trial sites gaining verbal consent from potential participants at the home assessment visit to be contacted by the evaluation team, and this did not systematically happen.

Sixty-one people who did not enter the trial after the initial home visit were telephoned after receiving an information sheet and letter inviting them to take part in a one-off interview to explore their current health or social care arrangements as well as their reasons for not taking part in the trial. Nineteen people agreed to take part and a further three who withdrew from the trial after joining the intervention arm ^ii^. Forty-two out of the 61 declined to take part in the qualitative study when telephoned. Despite declining to be interviewed, most people offered some information regarding their reasons for declining to take part in the trial during the telephone call and agreed that the information could be recorded: 8 stated that they (or the person they cared for) were too ill or incapacitated; 8 did not understand the intervention and why they were being offered to join the trial; 11 did not think they needed TH or TC and/or did not want it; 3 thought the trial was too disruptive; 6 cited personal reasons (e.g. going away, death of a family member, work commitments) for not taking part.

Those agreeing to be interviewed for the qualitative study signed a consent form prior to their interview. The majority of participants came from the east London site of Newham (13) where staff more readily obtained consent to be contacted with a view to being interviewed (see table 1 for details of interview participants). With additional recruits from Cornwall and Kent, we were able to achieve a wide variation of participants with the various health conditions as well as social care needs (see table 1). The mean age of respondents was 71 years and the majority of respondents were men (n = 14). In 4 cases the interviewee was the carer(s) for the person invited to join the trial, and in a further 5 cases, a partner or carer contributed to the interview. Whilst the sample was opportunistic, we ensured that we continued to follow-up willing participants until we reached saturation in the data analysis, and we conducted interviews iteratively, whereby the analysis of initial interviews was used to inform further collection.

**Table 1:** Participant details

**ID**	**gender**	**Age**	**Index condition for telehealth (TH)**^‡^**or telecare (TC)**	**Site**^**†**^	**Carer/partner present**
ID4 ^α^	M	72	Diabetes	N	
ID32	W	35	TC	N	Y* (both parents)
ID27	W	78	Diabetes (COPD)	N	N
ID31	W	61	Diabetes	N	
ID33	M	66	COPD (HF)	N	Y
ID34	M	69	HF	N	
ID133^α^	W	67	TC	N	
ID5 ^α^	M	70	HF	N	
ID25	W	89	COPD	N	Y
ID28	M	85	COPD	N	Y
ID29	M	68	Diabetes	N	Y
ID35	M	69	Diabetes	N	
ID136	W	92	Diabetes (arthritis)	K	
ID134	M	70	COPD (lung cancer)	K	Y*
ID135	M	82	HF	K	
ID161	M	23	TC	K	Y*
ID160 ^α^	W	64	TC	K	Y*
ID146	M	71	Diabetes	K	
ID145	M	90	HF	C	Y
ID156^**§** α^	M	85	COPD	N	
ID92^**§**^	M	73	Diabetes (heart and lung problems)	K	
ID89^**§**^	W	83	Diabetes	K	

^‡^ Additional conditions as described by respondent in brackets.

^**†**^ N = Newham, K = Kent, C = Cornwall.

* Interviewee was carer(s) alone.

^**§**^ Withdrew from the trial.

^α^ Had formerly immigrated from countries in South Asia, Africa and Eastern Europe; these participants spoke English as a second language.

Interviews were conducted by CS and RB and included topics such as the history of health and social care problems, previous and current care arrangements, perceptions of the equipment and the trial, expectations of the intervention and the potential impact on management of health and care needs. With the exception of one respondent ^iii^, interviews were audio-recorded and transcribed. Transcript data and field notes were organised with aid of Atlas.ti and the data were analysed thematically using some of the techniques of a grounded theory approach including constant comparison whereby we compared the data across cases to elicit common key themes and unusual cases [30]. Analysis of qualitative data often falls into three stages: data reduction, data display and conclusion drawing; and variations of a grounded theory approach tend to echo these three phases via open coding (data reduction), axial coding (data display), and selective coding (conclusion drawing) [31]. Three members of the team (CS, RB, AR) independently coded interview data using an open coding technique in Atlas.ti and each member of the team entered codes into separate versions of an Atlas.ti hermeneutic unit. This process enabled team members to become immersed in the data and develop early descriptive and brief codes (e.g. access to services, care routines, relationship with GP, use of technology). A number of early codes represented secondary constructs derived from previous research on chronic illness experience including ‘biographical disruption’ and ‘coping’ [32]. These initial codes were discussed at regular team meetings in order to develop and refine the initial codes into key themes. As part of this process we wrote notes and memos to move from the simple descriptive codes to more critical codes and three over-arching themes which are outlined in the results section of the paper. The whole approach to analysis reflected elements of both deduction and induction because we had some preliminary ideas about factors that might influence decisions to decline participation (based on previous research in the field), but we were also open to being surprised by the data in order to allow new and unexpected findings to emerge. This is commonly the case in grounded theory approaches which tend to entail a constant interplay between induction and deduction [33].

In keeping with an iterative approach common in qualitative research, analysis of the initial interviews informed subsequent interviews and prompted additional observational data collection from home visits where the intervention and the trial were explained as part of the consent process. This was because early respondents indicated a lack of understanding about the equipment and the trial when describing the initial visit when information was imparted. Observational work was conducted by CS and RB, and comprised shadowing of 8 members of health and social care staff (1 or more in each site) conducting home visits to 23 people (additional to the people interviewed). Field notes were made of observational visits and explored thematically alongside interview data. The data presented in the paper are taken primarily from interviews but with several illustrations from the observational data within each of the themes.

## Results

Analysis revealed a number of factors contributing to decisions to decline or withdraw from the trial within the following themes: requirements for technical competence and operation of equipment; threats to identity, independence and self-care; expectations and experiences of disruption to services.

### Requirements for technical competence and operation of equipment

Many respondents discussed their decision to decline the trial in the context of views about the technological nature of the equipment. It seemed unsurprising that some people who felt they would have difficulty engaging with the technological requirements of the equipment, had found other forms of technology to be problematic. For example, the following respondents reflected on the place of technology in the modern world and the generational differences regarding the use of contemporary communication technologies:

"‘When you have a hassling day; I stood at my front door the other day and I thought, 'really, truly, this world's not for me now, it's too complicated,' … you don't speak to anybody now, you get buttons you push and press and, just a nightmare… I've got a mobile phone but it’s emergencies… if I want my daughter, that's all and I wouldn’t even know how to use it. I've got instructions.’ (ID27)"

"‘The older you get the more forgetful you get, it's sometimes difficult to manage that sort of machinery, isn't it, to remember how to do it … younger people obviously that are computer wise… key boards, on the key pads and all the texting and everything… I think when you are not used to it … you need to read the manual every time you want to do something.’ (ID33_wife)"

Participants’ reflections on the place of contemporary communication technologies and their sense of alienation from such technologies were often combined with doubts about their capacity to engage with what they understood to be the operational requirements of the equipment. The following respondent in the east London site, who spoke some English as a second language, explained his decision to decline the trial as being due to a combined lack of confidence with language and a lack of confidence with technology. This was an important issue because of the ethnic diversity within the Newham population where the equipment was only being provided to operate in English:

"‘Because I am not much good English to read the computer, you know that …see you know what thing you are going to type and… the gentleman show me the thing… that one thing connect with your television and weight machine…one with the computer, with the internet… I think if I try I can, but depend on the internet using you know, I don't know how to use the internet.’ (ID4)"

Uncertainties regarding the technological aspects of the equipment did not seem to be mitigated by explanations presented at the home assessment visit. For example, the above respondent was not alone in thinking (following the home visit) that he would need to ‘type’ on a keyboard, when in reality no typing was involved, nor was there any need for familiarity with using a computer or the internet. Other respondents indicated a similar misunderstanding.

Comments made in interviews following the home visit indicated a high level of uncertainty regarding the technology being offered and that staff varied in their own knowledge of the intervention:

"‘They didn't show, didn't show me any actual um, equipment, but they mentioned [it] worked in conjunction with the television or PC or something like that, or a mobile, and I don't have either… I got the impression from what he said that er, being as though I didn't have those… the help I'll be able to get, would be sort of, rather limited… I mean, I'd have another, just under another seven years to wait before I got my free license.’ (ID34)"

Perceived uncertainties and questions that had remained unaddressed from the initial visit sometimes led to the respondents asking the interviewer more questions about the intervention:

"‘Well, he said [staff conducting home visit] you can … for monitoring your own blood pressure and… He said, ‘I don't understand anything about it.’ He was quite a nice chap, but… So I've got to be told to use a computer?… can I ask you, what is the technology?’ (ID25)"

The three participants who withdrew from the trial after they had received the equipment, described some technical difficulties they had in getting the equipment to work (ID156, ID89), and false alarms due to faulty readings (ID92). They described the response to some of these problems as being ‘slow’ and ‘frustrating’, and one described how the proposed solution (an additional phone line) brought further disruption:

"‘Nothing, wasn't working at all… And the next thing you know he comes in and he says, 'you can't do without the… phone line,' and so I said to him, 'It is somewhat of an aggravation, you must take it back.'… I couldn't, you know, have too many wires here and all this, you know. I said to myself, 'why should I have it?'’ (ID156)"

Observations of the home assessment visits added further examples to illustrate the findings within this theme from the interviews. For example, field notes demonstrated that people could easily misinterpret the technological aspects of the intervention as they recorded the ‘blank’ expressions on faces of some potential participants when staff verbally described the interventions and used terms such as ‘broadband’ ‘bluetooth’ and ‘internet’. At one home visit, the staff member conducting the visit explained to the potential recipient of telehealth that the recordings from different pieces of equipment were sent from the telehealth unit to the monitoring centre via an internet connection. The member of staff explained that an adapter would be fitted to their phone connection with permission in order to provide access to the internet. The patient then asked if she would be able to get lots of information from the internet and seemed to assume she might be able to use it for general purposes. However, she also went on to state that she was not at all certain she wanted to take part because she did not think she would know how to use it and because she did not think it would help.

Observation visits in the three sites also recorded views from staff that they had restricted time in which to explain the interventions because they were often required to conduct 8 or 9 home visits each day. As one member of staff stated, she always tried to explain the equipment but often met with very ‘blank faces’. On the day she was shadowed to conduct home visits, this particular member of staff had 9 patients to visit. At the first appointment, which was for a potential recipient of telecare interventions, she did demonstrate use of a ‘lifeline’ pendant which was to be worn round the recipient’s neck, enabling them to push an emergency button. All of the potential telehealth participants were shown a laminated information card with pictures to demonstrate how the telehealth equipment worked. In several cases, patients were also shown a piece of peripheral equipment such as blood pressure monitor.

### Threats to identity, independence and self-care

Most respondents indicated that they associated the use of telehealth and telecare with a high degree of dependency and ill health. In the majority of cases, respondents seemed to want to distance themselves from negative connotations of old age, sickness and dependence, and instead depicted themselves as having a strong sense of personal responsibility for maintaining health, self-care and independence that could be threatened by the interventions:

"‘I said to the man [who came to do the home visit], "I appreciate what you're doing… but I'd feel more crippled"… As long as I can get out, that's all I am worried about. As long as my feet keep going, I'll be alright. Yeah. I mean, sometimes we're out shopping and might see these elderly people - we're old; eighty four years old, the both of us are. We see these old people… hobbling along… and we're walking, you know, and think I wonder what age they are.’ (ID28)"

The respondent below also talked about the need to keep going and getting outside rather than monitoring his health indoors:

"‘You've got [to have] the will power… if I can't do it I am finished. If I wouldn't have that I'd be, I'd be stuck inside here you know, and looking through the window like… I throw myself in the garden and everything. Everything I do I'm working on, I cook myself dinners and everything.’ (ID156)"

Further examples illustrate the perception of TH and TC as appropriate for someone who was ‘a lot more ill’ (ID31) or if there was ‘no-one in the house’ (ID35). Responses commonly indicated a strong sense of personal responsibility for health, illness and self care; and the interventions threatened to undermine such a sense of ‘control’ and current approaches to managing health problems:

"‘I think you feel like you're not in control of your life… I just felt that, well, it certainly wasn't for me, and to, from how he explained it, um, you tended to have to do your blood test every single day… I try to be a bit more relaxed and… I just felt it, it did put a bit more pressure on me… you know, holidays or if I had to stay at my mum's, oh God, I've got to come home and do the machine.’ (ID31)"

For the above respondent, a sense of control was maintained by keeping her health problems within a broader perspective alongside other priorities in life, such as her caring responsibilities for her mother and grandchildren. In this way, her diabetes was allowed to fade into the background of her life. The following presents another similar example for this man who originally came to live in the UK from Ghana. He was planning to do some traveling, and whilst he talked about his active self care strategies, he was also keen to keep ‘illness’ in perspective, and indicated a degree of ‘chance’ was also involved:

"‘For me I see there are two things in life, the day you were born and the day you will die, you have no control, within it yes, you keep yourself healthy. That is all. So, I don't drink, I don't smoke, and I am a vegan so… If you live in this world and then you start complaining… thinking about things alone, well will make you, you know, it is better you don't live at all… I am not worried… if death comes today ‘bye, bye’ I'm gone!’ (ID5)"

In the following extract, a respondent talks about how the intervention would increase his dependence on his wife who would be required to help him to use the equipment because he was blind. This would be in marked contrast to the level of independence he had established in managing his condition:

"‘It would rely upon my wife to input the information via the TV [due to blindness]. And she doesn't like technology one bit… I couldn't do it myself. You know, I have a job to read the blood sugar, I have a job with my blood pressure. All the things that I do for myself are easy; I make my own life easier you know. I've got a telephone with the big numbers.’ (ID29)"

Other respondents were also keen not to dwell too much on their illness, preferring not to be reminded if their recordings were abnormal because it would make them worry (ID35), or remind them that they were not ‘behaving’ (ID89). One man preferred to distance himself from medical details (ID135), and some viewed the intervention as having the potential to make them ‘hypochondriac’ (ID33P, ID145[wife]). This was also illustrated in one of the cases observed whilst shadowing the staff conducting home visits. Following the description of the equipment, the potential recipient (ID_HV5) said to the member of staff *“I won’t lie to you, I don’t want to do it”*. He went on to explain that he did not want attention focused on his health problems and his aversion to receiving too much information about medical matters (field notes ID_HV5). Similar to other interview respondents, he also seemed surprised to be offered the intervention and stated *“we’re [referring to himself and his wife] not old enough are we?”* (field notes ID_HV5).

Others viewed the interventions as posing a threat to independence and activity, for example, by inducing ‘laziness’ (ID34). The following respondent seemed to indicate a precarious level of independence because of great difficulty with daily tasks due to additional problems with arthritis, and thought the equipment might further undermine her level of activity and independence:

"‘Because my hands were very bad at that time. They were so very, very swollen and hurt like mad … Well, I think I did say with my hands, I couldn't cope with anything fiddly… It's not because I don't want to necessarily. But I feel like as I say, I lost a lot of my confidence. For instance, I wouldn't be able to go on a bus tour or anything like that anymore.’ (ID136)"

In 3 cases potential recipients of the intervention were considered *too ill* or *too dependent* by their carers, and their views highlighted the importance of the identity and role of carers. These respondents considered the family member they cared for to be in need of a level of human care and supervision that was impossible to provide via telehealth and telecare equipment. For example, in one case the carer of a man receiving palliative care for lung cancer did not perceive the intervention to be helpful because someone was always with him and he was already being carefully monitored at home by community and hospice staff (ID134P). In two other cases, respondents had turned down the offer of telecare on behalf of their adult children who had learning disabilities and whom they had cared for all their lives:

"‘Well that's the reason we turned down the tele thing, because somebody needs to be with Jane all the time… So if you’re with somebody all the time… you know, exactly if there's anything wrong, just by their behaviour.’ (ID31_Father)"

"‘It wouldn’t be beneficial; we would have call outs all the time… he [son] is very poor at taking messages off the phone… they would have been at the door and even if he answered the phone, he would sound as if he was in trouble when he wasn't.’ (ID161_Mother)"

### Expectations and experiences of disruption to health and social care services

A final strong theme across many of the interview accounts related to potential changes in service provision that might arise from having equipment installed, and this was reflected upon in relation to current and previous experiences of services. Respondents often described their satisfaction with current services and how they preferred the existing care relationships that they had with health care providers:

"‘They put things in your home don't they… and it all goes through to somebody else. You know, you don't have to go to the doctors. It's all done indoors and all that. Too complicated for me… no, no, I just like things plain and simple. I'd sooner go over to the doctor. I mean, I can go over there if it's an emergency.’ (ID27)"

"‘But, as I say, I go to see the nurse for my asthma every couple of months, and if I don't she sends for me, and the doctor, he keeps an eye on my blood pressure. I go to, um, The London, er, once a year for my renal tests… So, we are, you know, we are completely under the care of the professionals.’ (ID28)"

Other respondents who had quite severe problems were often already receiving specialized services. For example, the following respondent with heart failure and COPD, described his relationship with a specialist care team:

"‘And then you have their emergency number, and then the minute you think your breathing is labouring and you've got a problem, you phone the red team, we've got the nurses mobile number… they come into your home… Every few days if you're bad, they can come back like every couple of days… it's absolutely brilliant.’ (ID33)"

His wife also talked about the value of dealing with specific staff who they knew well:

"‘I am perhaps a bit pessimistic about things like that, but I don't always have faith in things like that [telehealth] you know; I think it is far easier for me to pick up the phone and phone Sally and say… Jack's not well and she will l say, 'oh yes, I'll pop round,' and start taking or she will give me advice and I am speaking one-to-one with someone rather than something going through and you think have they got that? Is there someone at the other end?’ (ID33_partner)"

In the following extract the wife of one respondent who was receiving palliative care through the local hospice and community nurses described their current service arrangements:

"‘I think I'm still of the same opinion, purely because as I say, we have such good contact with our district nurses and our supporting teams around us. I mean, I've only got to phone the hospice and somebody will come out…we've got so many contacts around us.’ (ID134_wife)"

For one of the potential telecare participants, her parents described a long history of problems they had experienced within the social care system and how this had impacted on perceptions of the current changes being offered, at a time when they had started to feel settled and content with service provision:

"‘No, I mean, you see she was diagnosed when she was fourteen months; we seem to have gone from one thing to another to another. You think you settled for a couple of years and then they change everything… They had a social worker come around there she took three, nearly three nights interviewing us about Jane's needs, went back to the office put it in her drawers as closed file, because she was leaving and that was the end of it… we lost faith in them.’ (ID32_father)"

Some respondents referred to the lack of integration of the trial with routine care and expressed surprise that doctors they consulted were not always aware of the trial (ID92). The following respondent described how this did nothing to encourage her to take part:

"‘I did notice, when I went to the GPs the other day…there was a note on there… but he didn't mention it… I think, you know, if they want to make more of it, then they've got to liaise with each other a bit more… because… if one of those people were to talk about it, it's a bit different, isn't it, than speaking to someone completely new.’ (ID31)"

Observations of the home assessment visits indicated that most members of staff made attempts to highlight that the research was being conducted via general practices and as one member of staff stated, she tended to start her visit by saying ‘your GP has put your name forward as someone who could benefit from telehealth or telecare’. Despite this, some people visited were also uncertain as to whether it would influence existing services. For example, one woman asked whether the use of telehealth would change her existing management at home where she had a carer for 1 day each week and where she said ‘on the whole, I manage pretty well’ (ID_HV1).

Two respondents who withdrew from the trial described how the service changes they experienced caused additional stress. For example, one woman said she *‘did not want to be a nurse’ (ID89)*, and the requirements to conduct daily monitoring were *‘too time consuming and frustrating’*, and she was much happier to have returned to a regular appointment (fortnightly) with the community matron. Another man (ID92) described the good care he received prior to joining the trial, but how he was subsequently discharged from the specialist professionals who had been involved in his care. Whilst he was entered into the trial for his diabetes, he described his main problems as *‘complex problems with my heart and breathing’*, and that the faulty recordings and changes in service provision were causing him great stress.

## Discussion

This study demonstrates that reasons for declining the possibility of using telehealth and telecare equipment for those invited to join the trial were often explained in terms of potential threats to existing self-care, independence and service arrangements, as well as concerns about competency to operate equipment linked to general views about contemporary technologies. Additionally, insufficient information and discussions about expectations regarding the interventions contributed to decisions to decline or withdraw from the trial.

Perspectives on health, self-care and dependency associated with decisions to decline the trial indicated that technology had the potential to define their health problems as something more serious than they felt it was or should be because it was stereotypically associated with being very sick, very old or highly dependent. This was even the case for some respondents with severe symptoms, and where people indicated a perception that TH might speed up an inevitable process of decline. Previous research demonstrates that people are often keen to distance themselves from negative stereotypes of ill health and ageing [34], and that health-related interventions, including telehealth and telecare, can be perceived to undermine identity and autonomy, especially amongst older people [19,20,35]. This study demonstrates how anticipation of such negative effects can impact upon rates of adoption for these interventions. However, whilst the majority of respondents in this study depicted themselves as *too healthy* and *too independent* for the interventions to be of value, the small number of respondents who depicted the people they cared for as *‘too sick’* or *‘too dependent’* demonstrate that these interventions can end up falling between two stools. This underscores the deep ambivalence that can be found towards home-based health technologies [17], and the varied perceptions about whether telehealth and telecare interventions should be used as a preventive strategy or a mechanism for crisis management that tend to be context dependent [36]. This implies that attempts to integrate these technologies across all levels of need, from those who predominantly self-manage, to those who require complex case management, would require a degree of cultural shift in the way such interventions are perceived. It also implies that interventions should be tailored to ensure they will fit in with life circumstances and individual approaches to self-management because one intervention is unlikely to suit all people with the same long-term condition. In addition, the responses from some carers suggest that these devices have the potential to undermine important aspects of care with implications for caring roles. This highlights the importance of carers’ perspectives when evaluating such interventions, and the need for a wider range of programmes to address carers’ diverse needs [37].

Whilst previous concerns have been raised about violations of privacy via telehealth and telecare interventions, the respondents in this study seemed to be less concerned with privacy, than with the psychological effects of monitoring that might make them focus on or become over anxious about their health. Many accounts indicated that part of their existing approaches to self-care included a desire for their long-term health problems to fade into the background within day-to-day life, and responses reflected their busy and active lives. Thus, they did not feel comfortable with the time pressures and disruption that equipment might bring, and some felt uneasy about the effects of intensive monitoring. Previous studies have described the challenges brought by the new ‘work’ and scrutiny associated with such interventions [12,38,39] and where the disruption of normalised everyday practices and interactions regarding management have been recognised a major barrier to the integration of e-health interventions into routine care and management [13,40,41]. The current study shows that anticipation of such changes can prevent people from trying out these interventions in the first place. This finding resonates with the analysis of May et al. who suggest that poor uptake of and adherence to interventions for complex chronic conditions are often due to the heavy burden on patients and the lack of congruence with patients’ perspectives on care [42].

It was evident that many respondents were somewhat ambivalent about using the technology to begin with because all of them had agreed to have a home visit with a view to possible installation of equipment within the trial. However, discussions about decisions to decline the trial indicated that concerns about competency to manage technology, or concerns about costs were sometimes compounded or left unaddressed at the home visit. This finding resonates with previous qualitative research within trials highlighting the importance of how information is imparted and discussed can have a major impact on recruitment [12].

Comments regarding current services indicated that respondents were reluctant to disrupt services that were currently working well and that they often valued highly. Much of this satisfaction seemed to be associated with stable relationships that had been developed with specific service providers and there were concerns about what changes the equipment might bring to those relationships. These concerns were borne out in those who had withdrawn from the trial after receiving the devices. They experienced service changes that they found difficult to cope with. These views and experiences highlight the importance of organizational context, and indicate that telehealth and telecare interventions might be more readily adopted if they are introduced as an integral part of routine service provision; where existing relationships with service providers are not disrupted and can be maintained alongside use of equipment.

All the above points indicate potential avenues for future research. For example, there is a need for further research to investigate who specific interventions work for and in what circumstances. Additionally, there is a need for further research and development to ensure designs of telehealth and telecare fit with the needs and preferences of service users. This in turn would be likely to improve adoption of interventions because patients and carers would be more likely to want to use them. An additional aspect that warrants further research is the degree to which TH and TC interventions impact on the quality of care experienced by patients and carers.

### Strengths and limitations

One of the limitations of the study is that it is based on a small group that is primarily a convenience sample due to the difficulties in recruiting people who declined the trial. This means that the study could not fully examine the contextual and organizational factors that might variably influence decisions about refusal or withdrawal between all 3 sites. There were also only a small number of those who withdrew from the trial within this sample. It also needs to be recognized that this is a study within a randomized controlled trial and the trial itself may have had some influence on decisions to use telehealth and telecare. Within the context of a trial, the ability to encourage participation is restricted through the ethical constraints of a technology that as yet lacks firm evidence. Despite these factors, the sample does include a mix of men and women with both telehealth and telecare needs, and the strength of the study lies in the focus on a group of people who are not usually considered within a trial evaluation and who therefore represent an often hidden group with important viewpoints on the intervention focused upon. In this sense, the study offers ‘transferability’ of findings rather than ‘generalizability’ and is in keeping with the common goals of qualitative research, whereas the emphasis is placed on the latter in quantitative research [43].

## Conclusion

This study raises important issues with policy implications in an era where there is an increasing emphasis on implementing telehealth and telcare interventions for supporting self-care for those with long-term conditions and with social care needs [44]. Of particular interest is the finding that such interventions are often considered as a potential major threat to identity and existing management routines and service use for respondents. It was also apparent that even where respondents were somewhat ambivalent in contemplating installation of equipment, their feelings of uncertainty were not mitigated when the prospect of installation for the trial was discussed at the home visits. This indicates that more detailed information and time for discussion could be valuable when introducing these interventions for the first time. Given the assumptions about appropriate candidacy for telehealth and telecare evident amongst respondents here, and concerns about technology and service changes, it seems important for potential recipients to have the opportunity to discuss their expectations and additional common concerns about technological aspects of equipment and service changes prior to installation. Additionally, these findings suggest the need for closer proximity between innovation design and evaluation, so that critical insights might usefully feed back into design and implementation [11,13] ensuring interventions are *‘minimally disruptive’* for recipients [42].

## Endnotes

^i^ In some cases this was a member of health or social care staff and in other cases they were assistants who were specifically employed and trained to conduct the home assessment. ^ii^ One of these 3 withdrew only a couple of weeks after the equipment was installed. The remaining 2 withdrew after several months in the trial. ^iii^ This respondent did not want a recording to be made and in this case notes were made at and following the interview.

## Competing interests

All authors declare that they have no competing interests.

## Authors Contribution

AR, CS, PB and SN conceived and designed the study. RB and CS conducted the interviews and coded them with AR. SH and MC and SN collected and analysed quantitative data drawn upon in the study. All authors contributed to interpretation and prioritisation of findings. CS drafted the paper with AR and RB. All authors contributed to writing the paper. AR is the guarantor. The Whole System Demonstrator Evaluation Team (WSDET) contributed to periodic discussions of the data collected for this study during team meetings, and commented on interim documents produced during the study period. Members of WSDET additional to specified authors include: MB, LR, LS, JB, J-LF, CH, JD, JB, A S, HD, VMN. All authors read and approved the final manuscript.

## Pre-publication history

The pre-publication history for this paper can be accessed here:

http://www.biomedcentral.com/1472-6963/12/220/prepub
